# Radiology reporting in oncology—oncologists’ perspective

**DOI:** 10.1186/s40644-021-00431-5

**Published:** 2021-11-25

**Authors:** Elisabeta Valeria Spînu-Popa, Dania Cioni, Emanuele Neri

**Affiliations:** 1 Regionalspital Emmental, Burgdorf, Switzerland; 2 Department of Translational Research, University of Pisa, Master in Oncologic Imaging, Diagnostic and Interventional Radiology, Via Roma, 67, 56126 Pisa, Italy

**Keywords:** Medical oncologist, Radiologist specialized in oncologic imaging, Communication, Structured report, Standardized criteria

## Abstract

**Background:**

Structured reporting and standardized criteria are increasingly recognized as means of improving both radiological and clinical practice by allowing for better content and clarity. Our aim was to examine oncologists’ opinions and expectations concerning the radiologist’s report to identify general needs in daily practice and ways to improve interdisciplinary communication.

**Methods:**

A 19-question survey was sent to 230 oncologists from three different countries (France, Romania, Switzerland) identified on the online web pages of different hospitals and private clinics. The survey was sent by electronic mail with an online survey program (Google Forms®). All recipients were informed of the purpose of the study. The data were collected by the online survey program and analysed through filtering the results and cross-tabulation.

**Results:**

A total of 52 responses were received (response rate of 22.6%). The majority of the respondents (46/52, 88%) preferred the structured report, which follows a predefined template. Most of the respondents (40/52, 77%) used RECIST 1.1 or iRECIST in tumour assessment. Nearly half of the oncologists (21/52, 40%) measured 1–3 cases per week. On a 10-point Likert scale, 34/52 (65%) oncologists rated their overall level of satisfaction with radiologists’ service between 7 and 10. In contrast, 12/52 (19%) oncologists rated the radiologists’ service between 1 and 4. Moreover, 42/52 (80%) oncologists acknowledged that reports created by a radiologist with a subspecialty in oncologic imaging were superior to those created by a general radiologist.

**Conclusion:**

Structured reports in oncologic patients and the use of RECIST criteria are preferred by oncologists in their daily clinical practice, which signals the need for radiologists also to implement such reports to facilitate communication.

Furthermore, most of the oncologists we interviewed recognized the added value provided by radiologists specializing in oncologic imaging. Because this subspecialty is present in only a few countries, generally in large clinics, further training might become a challenge; nevertheless, intensive efforts should be made to enhance expertise in cancer imaging.

**Supplementary Information:**

The online version contains supplementary material available at 10.1186/s40644-021-00431-5.

## Background

With the exponential development of medical imaging modalities in the past few decades, the role of radiologists in the management of oncologic patients has constantly expanded, since the results of imaging modalities heavily impact not only primary diagnosis but also treatment guidance during the entire course of the disease.

An article published in the Journal of Clinical Oncology in 2019 raised awareness of the failure to deliver consistent, high-quality oncologic imaging services for most radiology practice models in the United States [1]. Moreover, according to a review conducted in 2018 by Schlemmer et al. [2], numerous studies have shown substantial rates of disagreement (13–56%) between initial cancer imaging reports and second-opinion readings by radiologists specializing in cancer imaging, with second-opinion reports indicating the need for change in patient management in between 13 and 53.5% of cases [3–8].

In the current clinical environment, in which both radiologists and oncologists are required to correlate and integrate an ever-growing amount of clinical, imaging and laboratory data, radiology reports are regarded as the key method of communication between radiologists and clinicians. Despite these changes, the style and format of radiology reports have generally remained unaltered for the last few decades [9]; in many cases, text-only qualitative reports are criticized because of numerous inconsistencies in their content, lack of structure and diminished comprehensibility of relevant information [10, 11]. Given this critique, it is worth considering whether greater standardization could allow for better communication and increased quality of radiology reports, thereby allowing fewer misdiagnoses.

A structured report with a template with standardized headings analogous to a checklist of necessary elements could provide an accurate, detailed and comprehensive radiology report, which should be better able to support clinicians in making therapeutic decisions [12]. The type of cancer, institution, and local setting in the oncology and radiology departments play a major role in the tumour assessment criteria and measurement techniques. Furthermore, standardized reports may support the clinician in assessing if a patient is eligible for clinical trials, changing patient management in this particular setting and facilitating data mining and data sharing in clinical or research activity [13].

The necessary technology is currently widely available, but despite its advantages, implementing a standardized structured reporting system may become a technical challenge. In the daily routine, fewer media-rich quantitative reports with tables or graphs are provided, and incorporating quantitative measurements and RECIST calculations into the radiology report may be viewed by radiologists as overly time-consuming [14, 15].

In this context, the purpose of this study was to examine oncologists’ opinions on the radiologist’s report, to raise awareness of their expectations, to identify oncologists’ perceived general reporting needs in terms of clarity and clinical usefulness in daily practice, and to indicate means of improving interdisciplinary communication so that oncologic patients can benefit from improved diagnosis, treatment planning and follow-up. A further aim was to explore oncologists’ opinion on the added value of a dedicated oncology imaging specialist in reporting and in multidisciplinary cancer team meetings (MDTs).

To improve patient access to oncologic imaging expertise, Nass et al. [1] highlight the need for leaders in the radiology and health care communities to find solutions designed to enhance expertise in cancer imaging, as subspecialization in radiology centres is currently specialized by organ or system, and cancer imaging is not formally recognized as a subspecialty in most countries, which may lead to a lack of intensive training or expertise in oncologic imaging.

## Methods

The survey was prepared by the authors based on their personal clinical experience following a literature review of selected medical literature [9, 12, 16] and according to published recommendations for internet-based surveys [13]. The questionnaire was drafted by the first author (E.P.) and shared with the other two authors to receive feedback and reach a consensus on the final form, resulting in a 19-question questionnaire (Table 1).

**Table 1:** Questionnaire for the oncologists about their opinion on the radiology service to oncology

#	Question text	Answer									
1.	Working place	a. Academic Hospital b. Nonacademic Hospital c. Private Practice d. other									
2.	How many years have you been practicing oncology?	a. I am still a trainee b. < 5 years c. > 5 years d. > 10 years									
3.	Your subspecialty (please select all that apply):	a. Head and neck cancer b. Urogenital c. Breast cancer d. Gynecologic cancer e. Gastrointestinal f. Pancreatic cancer g. Liver/ Biliary tract h. Lung cancer i. Melanoma j. Sarcoma k. Lymphoma l. Leukemia m. Pediatric oncology n. Other (please specify)									
4.	Regarding the use of in-house vs. external radiology services, how often do you use in-house services:	a. in less than 50% of the cases b. 50–80% of the cases c. I use only in-house radiology services									
5.	On a scale from 0 to 10, 0 meaning extremely unsatisfied, 10 extremely satisfied, how do you rate the overall service you receive from the in-house radiology department?	1	2	3	4	5	6	7	8	9	10
		○	○	○	○	○	○	○	○	○	○
6.	How easy is it for you to find tumour measurements in the radiology report?	a. every time very easy b. most of the time easy c. sometimes easy, sometimes difficult d. most of the time difficult e. I never/ almost never find the measurements.									
7.	How often do you, or your team measure tumours?	a. daily more than 3 cases. b. daily 1–3 cases c. weekly 1–3 cases d. monthly 1–5 cases e. every now and then, less than once a month f. never									
8.	Which measurement criteria do you currently use for tumour assessment? (please select all that apply)	a. RECIST 1.1 or iRECIST? b. Modified RECIST, for example: Choi for GIST, size and attenuation on CT (SACT), Lugano Classification, volumes, mesothelioma method. c. World Health Organization (maximum 10 target lesions, up to five per organ). d. Not applicable (I do not use tumour measurements for assessment) e. Other (please specify)									
9.	Do you find the text only radiologist report, with minimal quantification, adequate for making tumour assessments?	a. always b. most of the times c. sometimes, but not very often d. never									
10.	How would you like to have tumour measurements presented in radiologist reports? (please select all that apply)	a. In the report text. b. Hyperlinked text included in the report, linked to selected image slices (when clicking on the described measurement in the report, a link opens with the image showing that measurement) c. Tables d. Graphs e. Other (please specify)									
11	When the radiologist saves key images with tumour measurements in PACS, it makes finding tumour measurements easier (please select all that apply).	a. Strongly agree b. Agree c. Neutral d. Disagree e. Strongly disagree f. I am not sure what key images are or have not used them g. I prefer to have the series and image number for the finding written in the report and I look myself for that finding.									
12	In most of the cases, which of the previous examinations should be compared with the current examination? (please select all that apply)	a. Most recent previous b. Baseline c. Nadir/ best response d. None e. Other (please specify)									
13	A structured report following pre-defined templates has better content and greater clarity than conventional, non-structured report.	a. Strongly agree b. Agree c. Neutral d. Disagree e. Strongly disagree									
14.	How do you prefer the order of findings in the body of a radiology report? (please select all that apply)	a. Anatomic order, from superior (head) to inferior (pelvis) b. By examination region (head, neck, chest, abdomen, pelvis) c. List of individual organs or by organ groups (lungs, liver, pancreas, kidney etc.) d. The most important finding first and then the stable findings e. A combination of anatomic and most important findings or impression f. It does not matter g. Narrative paragraphs without lists or outline h. Other (please specify)									
15.	What should the radiologist report impression/ conclusion include? (please select all that apply)	a. Presence or absence of new lesions b. Target lesion measurements c. Disease progression, response or stability clearly stated d. Clinically significant, related findings e. Clinically significant, unrelated findings f. Recommendation for further evaluation and patient management. g. Other (please specify)									
16.	In most of the cases, a report made by a radiologist subspecialised by organ or system is preferable to a report made by a general radiologist (please select all that apply).	a. Strongly agree b. Agree c. Neutral d. Disagree e. Strongly disagree f. An added value of a subspecialised radiologist is only when participating in multidisciplinary cancer care teams									
17.	In your experience, a report made by a dedicated cancer imaging radiologist is of greater value as compared to a report made by a general radiologist.	a. Strongly agree b. Agree c. Neutral d. Disagree e. Strongly disagree f. An added value of a dedicated cancer imaging radiologist is only when participating in multidisciplinary cancer care teams									
18.	Do you have any recommendations for improving the radiologist oncology report? (optional)										
19	Your name (optional).										

The oncologists were identified after requests for sharing networks addressed to the author’s acquaintances (present and former colleagues) in the countries in whch the main author current practices (Switzerland) or could establish contact with oncologists (France and Romania), as well as by directly conducting a Google® search for individual e-mail addresses on the websites of different hospitals and private clinics in the aforementioned countries. The inclusion criteria in the study were medical oncologists, both specialists and doctors in training, who were randomly contacted, regardless of oncological subspecialty. A total of 230 oncologists from 38 different institutions in these three countries (France, Switzerland, Romania) were invited to complete the questionnaire. The survey was sent once by electronic mail through an online survey program (Google Forms®). All recipients were informed of the purpose of the study.

The survey included single-best-answer questions, as well as questions asking respondents to “Select all answers that apply”. All answers to the questions were marked as mandatory, except for the name of the respondent and a free-text question regarding suggestions for improvement or recommendations.

The data were collected and tabulated with an online survey program, but to facilitate statistical analysis, the answers were transferred to Microsoft Excel® (Microsoft Office®, 2019), in which data segmentation was performed, then filtering and cross-tabulation of data, with the objective of creating simple analyses and graphs. Readers may approximate the standard error of percentages shown by using the following formula: standard error in percentage = 100 × √*p*(1 – *p*)*/n,* where *p* is the proportion of participants with a certain characteristic, and *n* is the unweighted number of participants [17].

## Results

Between August and October 2020, 230 oncologists were invited to answer the questionnaire, and a total of 52 responses were received (a response rate of 22.6%). All responses received were included in the analysis.

### Respondent demographics

**Fig. 1:**
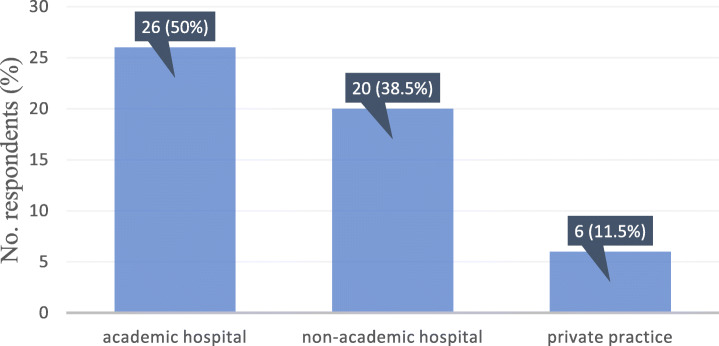
Bar graph of distribution regarding the working place

Half of the respondents (26/52, 50%) were from academic hospitals, 20/52 (38.5%) worked in a nonacademic hospital, and a minority (6/52, 11.5%) worked in private practice (Fig. 1).

**Fig. 2:**
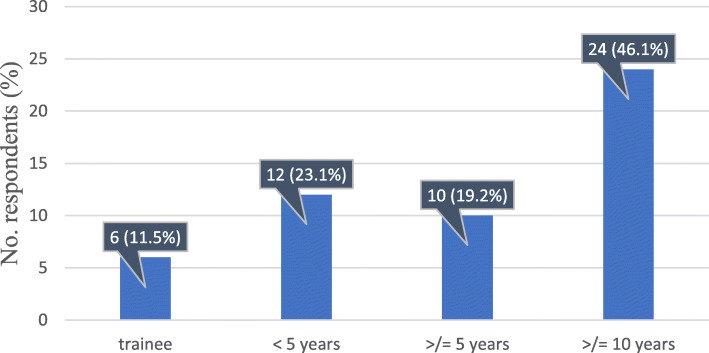
Distribution according to the level of experience of the respondents

Regarding years of experience, nearly half of the subjects (24/52, 46.2%) had been practising for more than 10 years, 10/52 (19%) doctors had been working between 5 and 10 years, 12/52 (23%) for fewer than 5 years, and 6/52 (11.5%) of the respondents were still in training (Fig. 2).

All the interviewed oncologists had more than one area of interest, and only 4/52 (7.7%) had no subspecialty. The clinical specialties spanned a variety of malignancies (Table 2), most commonly gastrointestinal (40/52, 76.9%); breast cancer (38/52, 73%); pancreatic cancer (36/52, 69.2%); and gynaecologic and liver/biliary tract malignancy (34/52, 65.4%).

**Table 2:** Summarized survey responses regarding the clinical subspecialties of the oncologists

Subspecialty	No. of respondents (%)
Head and neck cancer	16 (30.8%)
Urogenital	24 (46%)
Breast cancer	38 (73%)
Gynecologic cancer	34 (65.4%)
Gastrointestinal	40 (77%)
Pancreatic cancer	36 (69%)
Liver/ Biliary tract	34 (65.4%)
Lung cancer	32 (61.5%)
Melanoma	16 (30.8%)
Sarcoma	18 (34.6%)
Lymphoma	4 (7.7%)
Leukemia	0
Pediatric oncology	0
Medical oncology	4 (7.7%)

### Opinion on radiology service

Thirty-six/52 (69%) respondents used in-house services in 50–90% of cases, whereas only 2/52 (3.8%) subjects exclusively used in-house services, and 14/52 (27%) referred their patients to the in-house radiology service less than 50% of the time (See Supplemental Material SM 1).

**Fig. 3:**
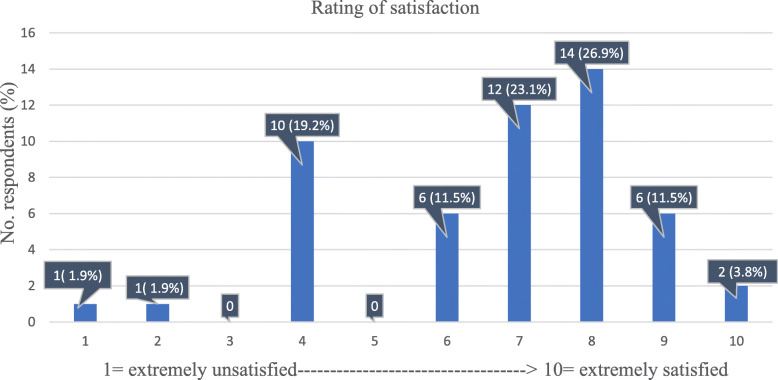
Graph shows the response distribution regarding the ratings of satisfaction of the oncologists with the overall service received from the radiologist

Oncologists’ overall levels of satisfaction with radiology services are shown in Fig. 3. On a 10-point Likert scale, 1 meaning extremely unsatisfied and 10 meaning extremely satisfied, for a considerable proportion of the respondents (34/52, 65%), the level of satisfaction ranged between 7 and 10. In contrast, 10 of 52 oncologists (19%) were quite disappointed with radiology services, evaluating it at 4/10.

### Tumour assessment

When asked about the difficulty of finding the measurements in the radiologist’s report, 18/52 (34%) answered that most of the time it was easy, 20/52 (38%) reported sometimes finding this information easily, 12/52 (23%) reported difficulty most of the time, and 2/52 (3.8%) could almost never find the measurements written in the report. None of the participants reported finding the measurements very easily every time (See Supplemental Material SM 2).

**Fig. 4:**
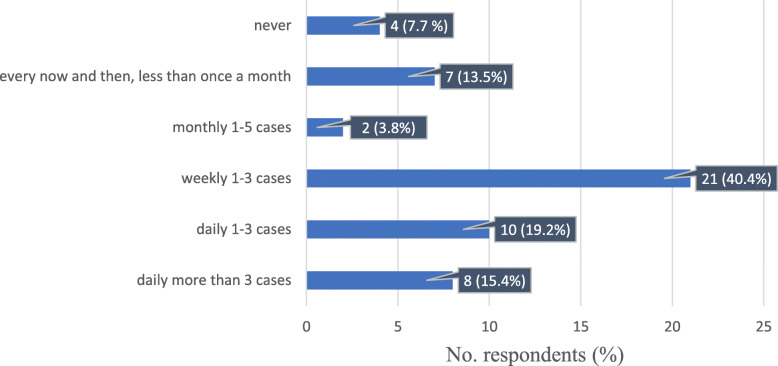
Response distribution of the frequency of measuring tumour burden

We were also interested in determining how often oncologists need to measure the tumour burden or have measurements taken. Nearly half of them (21/52, 40%) measured 1–3 cases per week, 10/52 (19%) measured 1–3 cases per day, 8/52 (15,4%) measured more than 3 cases per day, and 7/52 (13.5%) needed to measure every now and then. Only 4/52 (7.7%) never had to measure tumours (Fig. 4).

A comparison between the participants working in academic hospitals and those working in nonacademic institutions is shown in Table 3. A slightly larger proportion of oncologists working in nonacademic hospitals (45%) measured 1–3 cases weekly compared to those working in academic hospitals (34%).

**Table 3:** Summarized responses- comparison between participants working in academic vs. nonacademic hospitals

	Academic hospital No. of respondents (%)	Non-academic hospital No. of respondents (%)
How easy do you find tumour measurements in the report?		
Most of the time difficult	6/26 (23%)	4/20 (20%)
Sometimes easy, but sometimes difficult	8/26 (30%)	8/20 (40%)
Most of the time easy	12/26 (46%)	2/20 (30%)
How often do you, or your team measure tumours?		
Daily > 3 cases	4/26 (15%)	3/20 (15%)
Daily 1–3 cases	6/26 (23%)	3/20 (15%)
Weekly 1–3 cases	9/26 (34%)	9/20 (45%)
Measurement criteria currently used for tumour assessment		
RECIST1.1/iRECIST	23/26 (88%)	13/20 (65%)
Is the text only report with minimal quantification adequate for tumour assessment?		
Sometimes, but not very often	10/26 (38%)	8/20 (40%)
Most of the times	14/26 (53%)	6/20 (30%)
Saved key images with tumour measurements in PACS, makes finding the measurements easier		
Agree/Strongly agree	22/26 (85%)	14/20 (70%)
A structured report following pre-defined templates has better content and greater clarity than conventional, non-structured report.		
Agree/Strongly agree	23/26 (88%)	16/20 (80%)
A report made by a radiologist subspecialised in oncologis imaging is of greater value as compared to a report made by a general radiologist		
Agree/Strongly agree	21/26 (81%)	16/20 (80%)

**Fig. 5:**
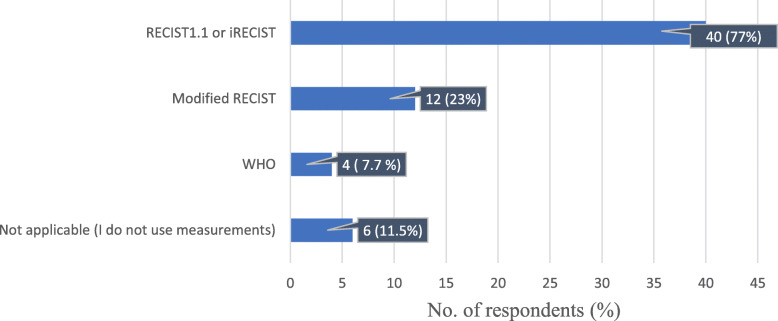
Distribution of the currently used criteria for tumour assessment

Regarding the currently used criteria for tumour assessment, most of the respondents (40/52, 77%) used RECIST 1.1 or iRECIST, while 12/52 (23%) used other criteria, such as mRECIST, Choi for GIST, size and attenuation on CT (SACT), Lugano Classification, volumes, mesothelioma method. Another 4/52 (7.7%) preferred WHO criteria, and 6/52 (11.5%) did not use measurements to assess response (Fig. 5). Eighty-eight percent of the participants from academic hospitals used the RECIST criteria, compared to 65% of the oncologists working in nonacademic hospitals (Table 3).

Most of the time, the text-only report was considered adequate for tumour assessment, according to 22/52 (42%) subjects. However, 20/52 (38.5%) found that this type of report was not often appropriate for tumour assessment, and in the opinion of 8/52 (15.4%) oncologists, the text-only report was never appropriate for tumour assessment. For a minority (2/52, 3.8%) of responders, the text-only report was always sufficient for assessment (See Supplemental Material SM 3).

When asked about assessing tumour response, 38/52 (73%) of the respondents preferred that the current examination be compared the most recent prior one, while 9/52 (17.3%) preferred it to be compared with the nadir, which is reasonable when a progressive disease is suspected, according to RECIST criteria. A total of 2/52 (3.8%) preferred the current examination to be compared with baseline, probably when a partial response is expected. A total of 3/52 (5.7%) oncologists answered that the oncologist should indicate in the order form which of the previous exams should be used by the radiologist for comparison (See Supplemental Material SM 4).

### Radiology report format

**Fig. 6:**
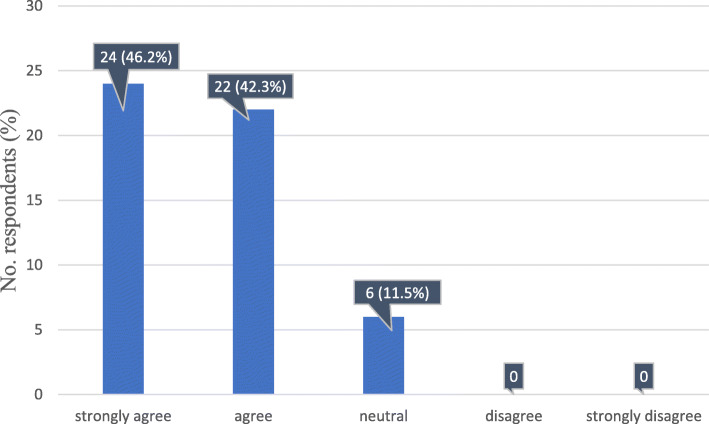
Response distribution regarding the statement: “Structured report, following predefined templates has better content and greater clarity than conventional, nonstructured ones”

In regard to the structured report following a predefined template, the majority of the respondents strongly agreed (24/52, 46%) or agreed (22/52, 42%) that such a format would greatly improve the quality of the content and bring greater clarity compared to the conventional, nonstructured report. Another 6/52 (11.5%) were neutral in this regard (Fig. 6). No significant differences were observed between oncologists working in academic and in nonacademic hospitals (Table 3).

Concerning the various possible ways of presenting the tumour measurements in the report, 34/52 (65.4%) respondents wanted the information provided in the report as written text, and 32/53 (61.5%) wanted a report with hyperlinked text linked to selected image slides. A total of 10/52 (19%) oncologists preferred tables or graphs to better assess the response (See Supplemental Material SM 5).

**Fig. 7:**
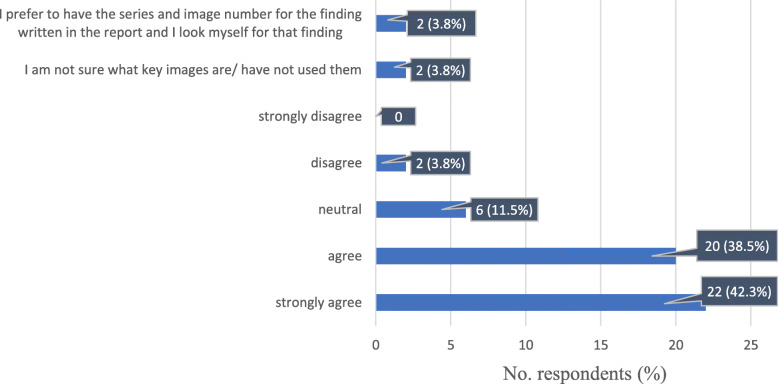
Response distribution for: “When the radiologist saves key images with tumour measurements in PACS, it makes finding tumour measurements easier

Most of the queried oncologists (42/52, 80%) found the key images saved by the radiologist in PACS helpful in assessing the tumour burden, while 6/52 (11.5%) respondents were neutral about this practice, and 2/52 (3.8%) did not find this approach necessary for assessment. A total of 2/52 (3.8%) oncologists did not know what the saved key images were or had never used that option (Fig. 7).

Most of the oncologists preferred to have the report organized either by examination region (head, neck, chest, abdomen, pelvis) (26/52, 50%) or in anatomic order from superior to inferior (22/52, 42%). Importantly, none of the respondents expressed a preference for narrative paragraphs without lists or outlines, and none chose the answer “it does not matter”.

According to the majority of the respondents, the report conclusion should include the presence or absence of new lesions, as indicated by 48/52 (92%) oncologists; provide target lesion measurements (46/52, 88.5%); and clearly state disease progression, response or stable disease (44/52, 85%). Only 20/52 (38.5%) oncologists found a written recommendation for further evaluation in the report’s conclusion helpful (See Supplemental Material SM 6).

### Opinion about subspecialized radiologists

Half of the oncologists strongly agreed (6/52, 11.5%) or agreed (21/52, 40.4%) that the quality of a report created by a radiologist specialized by organ or system is superior to that by a general radiologist. A total of 14/52 (27%) were neutral on this point, and a minority disagreed (4/52, 7.7%) or strongly disagreed (2/52, 3.8%). Of the 52 oncologists surveyed, 5 (9.6%) considered the added value of a radiologist subspecialized by organ or system to emerge only when participating in MDTs (See Supplemental Material SM 7).

**Fig. 8:**
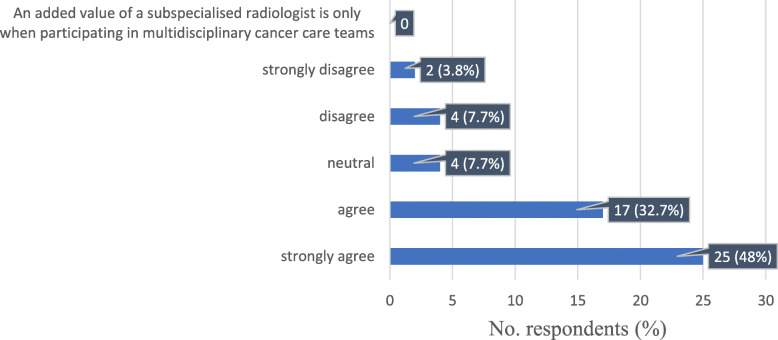
Response distribution for the statement: “In your experience, a report made by a dedicated cancer imaging radiologist is of greater value as compared to a report made by a general radiologist”

When asked about their opinion on the role of a dedicated oncology imaging specialist, the majority strongly agreed (25/52, 48%), or agreed (17/52, 32.7%) that the report by such a specialist is better than one by a general radiologist. A total of 4/52 (7.7%) were neutral in this regard, and only a few disagreed (4/52, 7.7%) or strongly disagreed (2/52, 3.8%). None of the respondents thought that the added value of a cancer imaging radiologist was observed only when participating in MDTs (Fig. 8).

## Discussion

The study highlights oncologists’ need for quantitative and objective reports, based mainly on RECIST criteria. As the results show, 88% of the participating oncologists preferred structured reporting over freeform narrative reporting. Moreover, a significant number of oncologists had difficulty in finding or interpreting tumour measurements in the radiologist’s report. As a result, up to 40% of the interviewed oncologists needed to remeasure 1–3 cases every week, either because they did not find the measurements in the report or because they did not agree with the radiologist’s assessment.

Although the RECIST/iRECIST criteria are intended for use in a clinical trial setting, oncologists increasingly rely on RECIST-based measurements when making clinical management and therapeutic decisions, mainly because they offer a simple way of measuring and communicating response assessment. The limitations of RECIST are also well known, thereby allowing clinicians to understand the pitfalls in selected cases. According to the study, 77% of the interviewed oncologists primarily used these criteria in their daily clinical practice. It is therefore mandatory that radiologists also understand and increasingly use RECIST in their daily practice to facilitate communication.

Questions #10 and #11 aimed to identify the preferred way of presenting tumour measurements in the report and the perceived benefit of the images saved in PACS by the radiologist. In our study, 80% of the participating oncologists found the images saved in PACS useful. Similar questions were used by Folio et al. in a study published in 2017 evaluating the opinion of oncologists about quantitative reporting in one institution, where the results were approximately the same, at 85.5% [18].

The purpose of questions #14 and #15 was to try to identify the most suitable format of a report, a concern also raised by other authors [18, 19]. The study once again highlights the usefulness of a standardized report in the opinion of oncologists. Radiologists should keep up with and use constantly evolving technology to their advantage to improve their efficiency, reduce errors and remain relevant.

Moreover, as has already been noted, there also is a need to expand patient access to cancer imaging expertise [1], and the study also highlights that the work of dedicated cancer radiologists is greatly appreciated and needed. The added value of a radiologist subspecialized by organ or system is well known and highly valued. However, most of the interviewed oncologists recognized the added value of a radiologist with a subspecialty in cancer imaging. Since subspecialization in radiology is currently generally categorized by organs or systems, and cancer imaging is not a formally recognized subspecialty in most countries, it is worth asking whether this method of training ensures intensive training and sufficient expertise in cancer imaging [1].

Although structured reporting is advocated as a tool to improve reporting in radiology and the ever-growing volume of data recommend the use of standardized criteria to improve interdisciplinary communication, to our knowledge, this is the first study aiming to examine the opinion of randomly chosen oncologists from different centres about the usefulness of structured reports, to rate their satisfaction level with the kind of reporting currently employed by radiologists, and to raise awareness of the need for enhanced training and expertise in cancer imaging, in the opinion of oncologists.

The authors are aware of the limitations of the structure of the survey and, ultimately, of the results of this study. Although low (22.6%), the response rate is similar to published response rates from online surveys [20–22]. The respondents included in this study were limited to several arbitrarily selected medical centres, and the number of participants was low; however, no significant differences were observed between the responders working in academic hospitals and those working in smaller institutions for most of the questions. In academic hospitals, oncologic patients are more likely to be included in clinical trials as compared to nonacademic institutions. As a potential consequence, a slightly greater proportion of oncologists working in academic hospitals use RECIST criteria compared to those from nonacademic hospitals. However, more than half of the respondents working in nonacademic hospitals (65%) still used these standardized criteria. Moreover, because a significant number of participants chose to remain anonymous, it was not possible to determine how many institutions are represented. The results may therefore not be representative of all cancer centres and are not meant to evaluate all radiologists.

## Conclusions

Although qualitative diagnosis still represents a large part of the clinical practice in radiology at this time, reliance on quantitative results and increased clarity in the radiologic reports are of great importance in oncologic imaging. The current study illustrates that structured reports and the use of standardized criteria in oncologic patients are much needed and appreciated by oncologists. Moreover, radiologists should continue saving images of tumour measurements in PACS, as this practice helps oncologists in their daily practice. Nevertheless, intensive efforts should be made to allow for the expansion of dedicated cancer imaging, for example, through fellowship programs, mentorship and routine participation in MDTs.

## Supplementary Information

**Additional file 1.**

## Data Availability

The dataset generated and/or analyzed during the current study are not publicly available due to the willingness of the authors to maintain anonymity of the participating colleagues that agreed to give their names for the survey. The database is available from the corresponding author on reasonable request.

## Abbreviations

MDT: Multidisciplinary cancer team meetings
RECIST: Response Evaluation Criteria In Solid Tumours
iRECIST: Immune Response Evaluation Criteria in Solid Tumours
mRECIST: Modified Response Evaluation Criteria In Solid Tumours
GIST: Gastrointestinal tumour
SACT: Size and attenuation on CT
WHO: World Health Organization
PACS: Picture archiving and communication system
