# Supplementary material for: Radiology reporting in oncology—oncologists’ perspective
Source: Cancer Imaging. 2021 Nov 25;21:63. doi: 10.1186/s40644-021-00431-5 (PMC8620527; doi:10.1186/s40644-021-00431-5)
Supplement: Supplementary file 1 — Additional file 1. [file 40644_2021_431_MOESM1_ESM.docx]

**SM 1. Distribution of the oncologists according to the frequency of using the in-house radiology service.**

**SM 2. Summarized responses for the question: “How easy is it to find the measurements in the radiology report?”.**

| Response | No. of respondents (%) |
| --- | --- |
| Never/almost never find the measurements | 2 (3.8%) |
| Most of the time difficult | 12 (23%) |
| Sometimes easy, sometimes difficult | 20 (38.5%) |
| Most of the time easy | 18 (34.6%) |
| Every time very easy | 0 |

**SM 3. Response distribution to the question: “How often do you find the text-only report, with minimal quantification adequate for tumour assessment?”**

**SM 4. Summarized responses for the question: “Which of the previous exams should be used for comparison?”**

| Exams used for comparison | No. of respondents (%) |
| --- | --- |
| Most recent previous | 38 (73%) |
| Baseline | 2 (4%) |
| Nadir | 9 (17 %) |
| Oncologist should indicate in the order form | 3 (6%) |
| Any exam | 0 |

**SM 5. Responses regarding the tumour measurements presented in the report**

| Exams used for comparison | No. of respondents (%) |
| --- | --- |
| Most recent previous | 38 (73%) |
| Baseline | 2 (4%) |
| Nadir | 9 (17 %) |
| Oncologist should indicate in the order form | 3 (6%) |
| Any exam | 0 |

**SM 6. Responses regarding the format of the body of the report and the content of the report conclusion.**

| Prefered order of the findings in the body of the report | No. of respondents (%) |
| --- | --- |
| By examination region (head, neck, chest, abdomen, pelvis) | 26 (50%) |
| Anatomic order from superior (head) to inferior (pelvis) | 22 (42%) |
| A combination of anatomic and most important findings or impression | 12 (23 %) |
| The most important finding first and then the stable findings | 9 (17%) |
| List of individual organs or by organ groups (lung, liver, pancreas, etc.) | 2 (3.8%) |
| Narrative paragraphs without lists or outline | 0 |
| It does not matter | 0 |
| Content of the report conclusion |  |
| Presence or absence of new lesions | 48 (92%) |
| Target lesions measurements | 46 (88%) |
| Disease progression/response/stability clearly stated | 44 (85%) |
| Clinically significant related findings | 27 (52%) |
| Clinically significant unrelated findings | 12 (23%) |
| Recommendation for further evaluation and patient management | 20 (38%) |

**SM 7. Response distribution of the oncologists’ perception about the reports and the added value of a subspecialized radiologist by organ or system when participating in MDTs.**
